# The Process of Self-Cultivation and the Mandala Model of the Self

**DOI:** 10.3389/fpsyg.2017.00024

**Published:** 2017-01-24

**Authors:** Meiyao Wu

**Affiliations:** Department of Education, National Kaohsiung Normal UniversityKaohsiung, Taiwan

**Keywords:** Hwang's Mandala model, Confucian education, self-cultivation, inner-outer harmony, psychology, teaching, learning

## Abstract

In his Mandala model of the self, Taiwanese scholar Kwang-Kuo Hwang sees each human being as a combination or intersection of private individual and social person, and also of knowledge and action. To further elaborate the model—with a particular emphasis on teaching/learning, the development of the ideal self and spiritual transcendence—this article will explore the psychological process of self-cultivation in the light of traditional Confucian thinking, which means keeping a balance between inner/outer and self/other. The Neo-Confucian thinker Zhongsha Mou's theories of “the awareness of unexpected developments” and his meditation/cognitive thinking opposition will also be discussed. The analyzed sources will include the traditional Confucian classics (the Four *Books* and *Liji, or Classic of Rites*) and especially the “*Lessons for Learning* (*Xue-Ji*)” in the *Classic of Rites* (*Liji*), along with the relevant textual research. Based upon a cultural-semantic analysis of these classics as well as of Hwang's central ideas, the author attempts to further conceptualize the process of cultivating the ideal self in Confucian education^1^.

## The concept of the self in western philosophy and psychology

In classical western philosophy, Plato is more focused on absolute ideas (ideals, “forms”) than on any concept of the “self”; his student Aristotle does talk about the soul (*psyche*) as the “form of the body” but still does not really talk about the individual self. In early modern European philosophy, of course, we have the seventeenth-century rationalist Descartes emphasizing the split between the individual *cogito* or “I think”—“I exist insofar as I am, at least in this moment, a thinking thing”—and the physical body^2^. The British empiricist Hume is more body-oriented in the sense that he says “we” are just a series of sense-impressions coming in from outside; our concept of a unified “self” is, like that of “things” and also of “God,” an illusion.

The German Kant, influenced by both, separates our rational mind, with it's *a priori* logical categories, from the act of perception and from the *Dinge-an-sich* (things-in- themselves) which lie beyond our perception and understanding. For Kant we do not “know” our self as an object of perception but presuppose it as lying “behind” all of our perceptions and thus our empirical knowledge. In twentieth-century philosophy we similarly have the split between Continental European “existentialism” (Heidegger, Sartre) and British analytic or empirical philosophy (Russel, Whitehead, A.J. Ayer). As for psychology, we have the Austrian Freud's psychoanalysis on the continental side and behaviorism (e.g., Skinner) on the British side, where Freud's theories, like the existentialists', may seem too “metaphysical” for the more empirically-oriented British thinkers. Kant's point that in one sense we do not “know” our self is arguably echoed with a variation by Freud's notion of the unconscious.

The “self”-concept naturally plays a significant role in western educational psychology (Purkey, 1988; Martin, 2007; Huitt, 2011). Freud's model of the psyche comprises three parts: the Id (*Das Es*), the Ego (*Das Ich*), and the superego (*Das Über-Ich*; Freud, 1923)^3^. Influenced by the self-concept of Freud among others, Erikson emphasized self-development and the self-identity crisis, and his follower Marcia's research also concerned the nature of our self-identity (Marcia, 1993; Erikson, 1994). Moreover, humanistic psychologists such as Rogers and Maslow regarded the self-concept and self-actualization as the core of their psychological theories (Maslow, 1954; Rogers, 1961). The “self” concept in western psychology originated from the rationalist and empiricist views discussed above, and from Hegel's more comprehensive notion of self-consciousness: our subject-object (observer-observed) consciousness forms our thinking and guides our behavior (Morgan, 1903). Self-esteem, self-regulation, and self-efficacy are derived from the ways in which the subject perceives the world around it and becomes self-conscious.

According to the Russian cultural-historical psychologist Vygotsky, the formation of human beings' higher mental functions was greatly influenced by their social-cultural-historical environment (Vygotsky, 1978). For example, the way Icelanders use language to describe “what is the feeling of cold” will be different from the way those who live near the equator use it to describe this. They grew up in different socio-cultural systems and also were influenced by different historical- semantic systems, and thus they will have different cognitive schema and linguistic symbols for describing “what is cold.” Similarly, psychologists who grew up in non-western societies might adopt different cognitive schema and symbols to express the self-concept and descriptions of self-development, etc. In traditional Chinese Confucian culture and society, there may be slightly different ways to describe or express things or concepts that in English would be called “the family” (*jya-ting*)^4^ or “benevolence” (*ren*)^5^. These Chinese terms/concepts suggest the ancient Confucian concept of “family” might include the “animals living under the house” as well as family members' activities in or around that same place or locus. This view, made clearer by the fact that ancestors were also seen as “extensions” of living individuals, is easily contrasted with the way modern western families may tend to see themselves.

This emphasis on the individual as an integral part of the family in more traditional cultures generally, then, and very clearly in ancient Confucian Chinese culture, is an example of how culture as well as language determines how we think, e.g., how we differentiate subject/object or use terms like “self” and “family.” In fact, in ancient (and also present-day) Hindu, Buddhist, Chinese Daoist and Chinese- Confucian thinking the subject itself can also be the object to be observed. That is, there may be no clear distinction between subject and object, and in ways that go beyond Hegel's notion of self-consciousness as the unity or mutual-embracing of subject-and-object (Cheng, 1991, p. 42, p. 70; Man, 2015, p. 2). Thus, in traditional South and East Asian thinking the constitution of the “self”-concept may be based on an inner reflection on oneself which, when seen as part of the whole educational process undergone by young people, is also called “self- cultivation.”

Indeed, in ancient Chinese culture there was a tendency to think of human life as a kind of open “field” that could be cultivated, and arguably the horizontality of this image may correlate with other forms of horizontality in ancient Chinese thinking. Most obviously, while western monotheistic religions—Judaism, Christianity, Islam—assume a radically disrupted vertical-transcendent model, according to which an all-powerful Supreme Being and Creator has total power over His creatures, the dominant “religion” in very early Chinese culture was arguably ancestor worship. In the latter, of course, the spirits or ancestors that are honored and remembered by their still-living descendants are hardly on a higher ontological level, transcending this immanent world like the Supreme Beings of western religions. Rather, they may still be immanent in this world, still “around” us—in our “family” or “house”—and accompanying us, so that rather than a bifurcated vertical or transcendent model we have a model, picture, or conception of “pervasive immanence.”

## Hwang's Mandala model of the self

Taiwanese psychologist and scholar Kwang-Kuo Hwang's Mandala model of the self—which has been influenced by ancient Hindu and Buddhist as well as Confucian thinking, and also by modern European thinkers such as the psychologist Carl Jung—not only pictures but actually already *is* a sort of highly-energized field, one which we are being invited to deeply reflect or meditate on, absorb, “cultivate.” Jung had adopted the symbol of the Mandala, a graphic or pictorial representation of “the center,” and used it to depict or represent the Self (Jung and Jaffé, 1963:20; Jung, 1964). According to his *Memories, Dreams, Reflections* (Jung and Jaffé, 1963), this Mandala includes or embodies the living experience of the collective unconscious of the human race. In other words, the center of the Mandala, as the Self, embodies the harmony and balance of the various opposed forces within the human psyche, which are influenced by its socio-cultural or collective life experience and values.

The Mandala symbol, which originates from the Indian religion and represents the universe, has been widely adopted by Buddhism. Especially after early Indian and Tibetan Buddhism were transferred to China, this symbol became gradually embedded in Chinese philosophy. It was well-known by the time of China's Song (960–1279 AD) Dynasty, when Confucian, Daoist and Buddhist thinking had already been integrated as Neo-Confucianism. The basic form of most Mandalas is that of a circle inside a square which is inside a still larger circle. From the perspective of Chinese philosophy, the mandala means or depicts the “field of an altar (*tan-chang*, 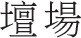)” and/or a field within which one's personality can be cultivated and guided toward perfect spiritual happiness^6^.

Partly influenced by Jung's and his followers' interpretations of the Mandala symbol in relation to the Self, then, and drawing upon his own research on the psychology of indigenous peoples, in which a major role is played by the individual's wider socio-cultural context, Kwang-Kuo Hwang also proposed a Mandala model of the Self (Hwang, 2015, pp. 87–88). This model also draws upon the ancient Chinese philosophical view of human life as an open field, the cultivation of which is really *self-cultivation* insofar as the self is formed or developed out of this wider field. For the Self taken as Person we may think of this wider field as one's family/community/society, while for the self-taken as Individual we may think of it also as the totality of one's own life experience, of one's own acts of experiencing life. Thus, these are two different directions or dimensions through which the Self may continue to be cultivated or perfected (Hwang, 2015, pp. 86–90)^7^.

According to Hwang's Mandala model, then, the Self is in the middle and there are four forces in the field that it needs to integrate and regulate in order to create a harmonious and balanced way of life (*dao*, 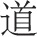). This way of life is dynamic, given both its location within the horizontal context of changing social interactions and its own vertical-temporal dimension. If the Self as Individual belongs to the psychological level, gathering, and regulating all of its experiences and its biological needs, the Self as Person belongs to the sociological level on which it serves as an agent-in-society, so that all of one's social roles—e.g., the roles of father, son, daughter, husband, wife, friends, co-workers—here come into play. If we also think of the Individual (as physical organism) as having a certain kind of relationship with (a certain awareness of) death and the cosmos, we may also think of the Person (as social organism) as having a certain but slightly different relationship with death and the cosmos (Hwang, 2014, pp. 38–39; Hwang, 2015, pp. 90–94).

**Figure 1:**
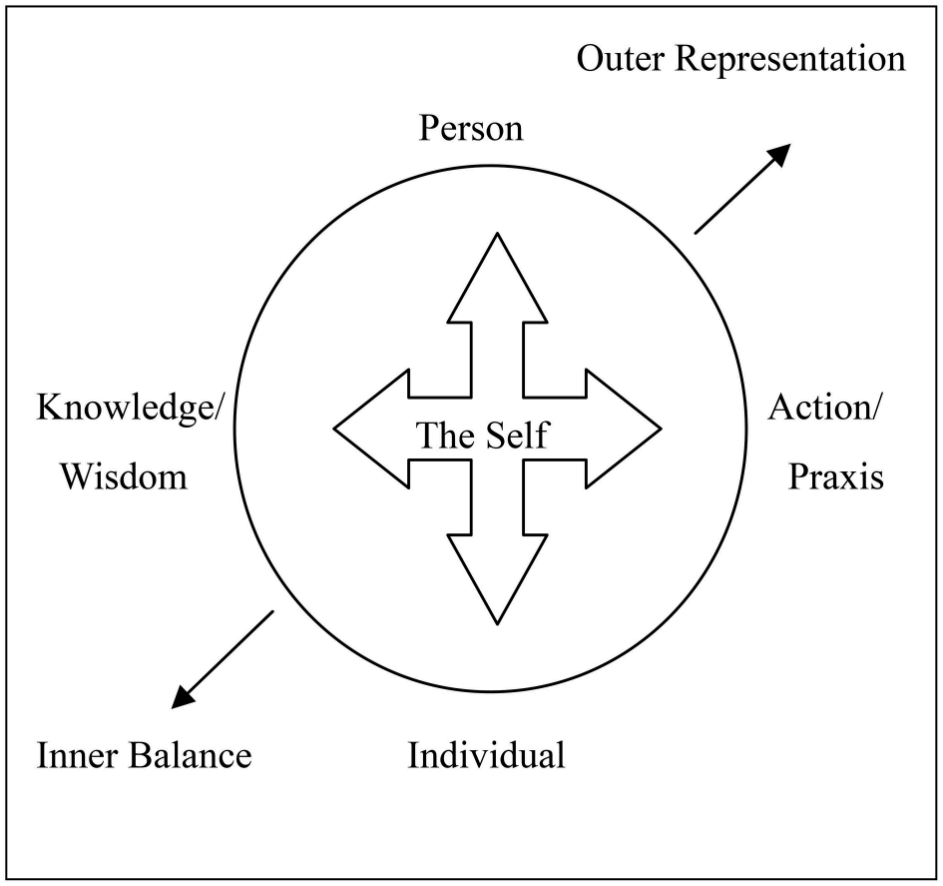
**Based upon Hwang's Mandala Model of the Self (Hwang, **2015**, p. 92; “Outer representation” and “Inner balance” were added to Prof. Hwang's Mandala model by the author)**. Mandala as symbol of the self and its realization in the context of Confucian social Relations.

Then we may see the horizontal line in the above model as extending from inner reflection (wisdom) to outer social action (praxis). The self is aware of the inevitable obstacles facing it, both in terms of its social roles within various contexts of social interaction and its biological needs and limitations. It absorbs the relevant knowledge based upon its own physical condition and social-historical-cultural background, and then transforms this knowledge into “insight wisdom” which it may act upon or project in the form of concrete social action or praxis. Here it would seem that Hwang is attempting to draw a universal model of the human mind on the one hand, and on the other hand a model that could describe the operation of the mind within the limits and under the constraints of various socio-cultural systems.

In other words, he is trying to elaborate an all-inclusive cultural theory which can describe not only the morphostasis (permanent or unchanging form or basis) of Confucian culture but also this culture's morphogenesis, its transformation within various East-Asian Confucian societies (e.g., China, Japan, Korea, Vietnam, Taiwan, etc.)^8^. This self-transformation is just as crucial as the self-cultivation that it follows from, or perhaps these two are virtually the same thing, two sides of the same process. Thus, if again we take into account the “field” of the self that is being cultivated, the Mandala has both spatial and temporal dimensions.

## A further elaboration of Hwang's Mandala model through readings of seminal confucian texts

### The confucian cultural value system: *Ren, Yi, Li, Zhi* in the context of existential relations

In the early Han dynasty (220 BC—9AD), the philosophical thinking of the hundred schools had already been gradually integrated into the mainstream philosophical schools of Daoism and Confucianism. Then, especially beginning from the Song dynasty (960–1279 AD), Daoism, Buddhism, and Confucianism were integrated into Neo-Confucian thinking, and so the Confucian value system still served as the foundation of mainstream Chinese culture and society, continuing to influence the common people's daily life.

In the Confucian classics or Four *Books*, especially in the *Analects* and *Mengzi, ren* (humaneness, benevolence), *yi* (righteousness), *li* (ritual, propriety), and *zhi* (wisdom) express the core values of Confucian ethics. In the *Analects, ren* cannot really be separated from *li* (ritual, propriety). *Ren* is the most abstract and universal moral principle as it is the principle of humanness in the sense of human interaction: the character *ren* (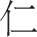) depicts “two persons.” Thus, in his *Lunyu* or *Analects*, Confucius says his “golden rule” is “Doing one's utmost (*zhong*) and putting oneself in the other's place (*shu*).” Here we see that the abstract moral principle of *ren* (humaneness, benevolence) must be solidified through the concrete social actions or forms, the cultural rituals of *li*^9^.

Then in the *Mengzi*, Mengzi takes *yi*-righteousness and *zhi*-wisdom as being in parallel with *ren*-humaneness and *li*-propriety^10^. When the *ren*-humane mind is represented or manifested *via li*-propriety, we may say that this manifestation is *yi*-suitable and *yi*-right, and so we have *yi* [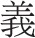]-righteousness. However, the process of representation or manifestation of inner *ren* as outer *li*-action is always complicated by the changing socio-cultural context(s). One needs knowledge and experience in order to properly understand this changing context, and this process of reflection and judgment is called *zhi* (wisdom). In the context of Confucian self-cultivation, then, not only must innate *ren*-humaneness be manifested in/as concrete, *li*-proper social actions or rituals, but *zhi*-wisdom must be used to judge which social actions are *yi*- righteous.

### The confucian self-other(s) duality and individual self-cultivation

The Individual dimension in Hwang's model, then, is related to the fundamental instincts and needs of human beings. In the *Liyun* chapter of the *Liji* (*Classics of Rites*) it is said that “The things which men greatly desire are comprehended in meat and drink and sexual pleasure; those which they greatly dislike are comprehended in death, exile, poverty, and suffering. Thus, liking and disliking are the great elements in men's minds (*Liyun* Ch.19)” (Legge, 1885)^11^. Here we have a clear duality of physical pleasure and physical pain or suffering, where these further imply the duality of “liking” and “disliking” on the psychological level.

In the *Mengzi*, there is a famous dialogue on the nature of human beings (or human being). Gaozi said that “to enjoy food and sexual desire is the nature of human being(s),” but Mengzi claimed that this is just one part of human nature. The difference, he said, between human nature and animal nature is that human beings have *ren*-humanity (benevolence) and thus a sympathetic or “commiserating mind”: “All men have a mind which cannot bear to see the sufferings of others^12^.” In other words, for Mengzi and the Confucian scholars this commiserating mind (*ren*-mind) is the foundation of the social order of Confucian society. Here we see that fundamental needs (e.g., for food and sex) are, in addition to being apparently more animalistic, are oriented toward *Individuals* (the self and the objects of its desires), while the *ren*-mind or benevolent mind has a humanistic orientation precisely because it has a *social* orientation—where here we may more likely think of Hwang's *Person*.

Here, however, the question arises: If both of these orientations (individual and social) may express the fundamental nature of human beings, then how does the outer (psychological) self-regulate the conflict between them in the inner psyche-self? We might want to set this question alongside one which arises from a passage in the *Liyun* of the *Liji* (*Classics of Rites*): “What are the feelings of men? They are joy, anger, sadness, fear, love, disliking, and liking. These seven feelings belong to men without their learning them” (Legge, 1885). However, if such emotions are natural to human beings, does not their proper expression still need to be cultivated? And if so, how—in the Confucian context—does this emotional education work?

In the *Zhong Yong* of *Liji* (*the Classics of Rites*) we have: “While there are no stirrings of pleasure, anger, sorrow, or joy, the mind may be said to be in the state of Equilibrium. When those feelings have been stirred, and they act in their due degree, there ensues what may be called the state of Harmony” (Legge, 1885)^13^. In other words, even when various confused or conflicting feelings begin to arise out of a completely peaceful or “empty” mind—where the latter term may seem to more easily fit Daoism and Buddhism, and the practice of meditation—in the Confucian context a well-cultivated Self or Psyche can still regulate and harmonize them.

In the traditional Confucian classics the teaching of poems and music was highly valued. Confucius said in the *Jinjie* of *Liji* (*The Classic of Rites*): “When you enter any state you can know what subjects (its people) have been taught. If they show themselves men who are mild and gentle, sincere, and good, they have been taught from the *Book of Poetry* (*Shi*). If they have a wide comprehension (of things), and know what is remote and old, they have been taught from the *Book of History* (*Shu*). If they be large-hearted and generous, bland and honest, they have been taught from the *Book of Music* (*Yue*)^14^.” As a humanist, Confucius believed that the cultivated man's emotional feelings will be well-expressed. He said that “The poem of *Guanju* in the *Book of Poetry* (*Shijin*) is expressive of enjoyment without being licentious, and of grief without being hurtfully excessive (*Lunyu* 3.20) (Legge, 1861)^15^.” Moreover, “In the *Book of Poetry* (*Shijing*) there are three hundred pieces, but the design of them all may be embraced in one sentence—‘Having no depraved thoughts’ (*Lunyu* 2.2)” (Legge, 1861)^16^.

In addition to the teaching of poems or literature, the Confucian teaching also emphasized music education. As the *Yuji* of the *Liji* (*The Classics of Rites*) said: “All modulations of the voice spring from the minds of men. When the feelings are moved within, they are manifested in the sounds of the voice; and when those sounds are combined so as to form compositions, we have what are called airs. Hence, the airs of an age of good order indicate composure and enjoyment (Legge, 1885)^17^. Confucius also said that “It is by the Odes (*shi*-Poetry) that the mind is aroused. It is by the rules of *li*-Propriety that the character is established. It is from Music (*yue*) that the finish is received (*Lunyu* 8.8) (Legge, 1861)^18^. In other words, the Confucian teaching emphasized literature, poetry and music education in order to *harmoniously* regulate the instincts and the natural feelings of human beings.

Another significant issue regarding the aspect of the individual is this: the physical life of the individual has temporal limits, and we are naturally conscious of these temporal limits and of the unexpected events which we will inevitably encounter in our lives. In the *Analects* Confucius sighed sadly when he heard that one of his students was dying. “The sickness is killing him,” he said. “It is the appointment of Heaven, alas! That such a man should have such a sickness! That such a man should have such a sickness! (*Lunyu* 6.10)” (Legge, 1861)^19^. In other words, in the real world there is no guarantee that a good man will be rewarded with good fortune; life is filled with unexpected developments. Then, how can one continually live well in the world?

According to the Confucian teaching, although our lives are limited by our physical, temporal and social situation, one still can take the time to create a meaningful life for him/herself. Such a life is of course constituted within the self-others context of our social interactions, where to some degree such interactions may be more predictable than spatial and temporal changes or disruptions. Thus, given our temporal (and spatial) limits, we should take the time to develop and sustain meaningful relationships with others, including our family members, partners, friends, neighbors, and co-workers. As Confucius says in the *Analects*: “While his parents are alive, the son may not go abroad to a distance. If he does go abroad, he must have a fixed place to which he goes (*Lunyu* 4.19) (Legge, 1861)^20^. Of course, creating a meaningful life through “education” or through “cultivating the next generation” are also regarded as significant ways of going beyond one's limited physical life^21^.

### Learning as self-cultivation of the person within the confucian social matrix

In Confucian relations, a person can play several social roles, e.g., those of father/mother, son/daughter, husband/wife, friends, superior/subordinate in the modern period (or monarch/courtier in the ancient period), etc. A well-cultivated person has to learn to manifest the proper behavior in various social-interaction contexts by playing various social roles. Here the problem is that of how to *judge* which kind of social actions are proper *(li*-propriety) or right (*yi*-rightousness) within given social interaction. According to Confucian ethical doctrine, the fundamental moral standard of *ren*-virtue or *ren*- humanity could be expressed or represented in various forms of *li*-proper actions within different socio-cultural contexts (Wu, 2013). In other words, *ren*-virtue could also be expressed or represented in various forms of social action. In the traditional Confucian classics, there are particular ways to cultivate a person who has the wisdom to judge and enact *li*-proper actions within ever-changing social contexts.

The main issue when it comes to the cultivation of persons is that of self-environment interaction. A person is born into a given society, one itself constituted by long-established historical-cultural forms with their own particular series of codes and social rituals. In the Confucian society, this means that there is a sum total of all *li*-proper forms which are based upon those moral principles of *ren*-humanity; the latter have been described by Hall and Ames as a “social grammar” or as the “underlying syntax of community” (Ames and Rosemont, 1998 p. 51; Hall and Ames, 1998).

According to the *Liji* (*The Classic of Rites*), teaching and learning the proper social behavior involve hierarchical steps, i.e., moving from the more to the less familiar in one's life experience, from simpler to more complicated social actions. As the *Xueji* (*Lessons for Learning*) said: “The learners could not transgress the order of study (imposed on them; Legge, 1885).” Thus, “as for the craftsman who repaired the ironware, his son should learn to sew a fur coat first, then he can learn how to put the soft hot materials on the ironware; as for the craftsman who produced the well bows, his son should learn how to weave the soft bamboo to make a dustpan^22^.” In other words, the learning process should follow the learners' basic capacities, and thus should proceed step by step. A similar description appeared in the *Xueji* (*Lessons for Learning*):

“If a student do not learn (at college) to play in tune, he cannot quietly enjoy his lutes; if he do not learn extensively the figures of poetry, he cannot quietly enjoy the odes; if he do not learn the varieties of dress, he cannot quietly take part in the different ceremonies; if he do not acquire the various accomplishments, he cannot take delight in learning. Therefore, a student of talents and virtue pursues his studies, withdrawn in college from all besides, and devoted to their cultivation; or occupied with them when retired from it, and enjoying himself” (Legge, 1885)^23^.

In other words, the learner should learn the basic knowledge and skills needed in various social contexts *within the same and similar contexts*, and then he can learn more complicated forms of knowledge and more complex skills.

The strategies of “modeling learning” and “learning by doing” are also widely adopted for teaching a person the various roles he or she may need to play in particular modes of social interaction. As the *Xueji* (*Lessons for Learning*) says: “The suitability of the lessons lies in their adaptability to circumstances; and the strength of the influence of examples lies in their relevance to the situation (Legge, 1885)”^24^. However, for beginning learners it may be difficult to fully grasp the spiritual force of the *ren*-humanity embedded in the various *li*-proper social actions, social relations or more generally the “social grammar.” Therefore, the need to learn the ethical principles by engaging in the relevant social activities (or social relations) is continually stressed by Confucian teaching. For example, in the *Analects*: “If a man withdraws his mind from the love of beauty, and applies it as sincerely to the love of the virtuous; if, in serving his parents, he can exert his utmost strength; if, in serving his prince, he can devote his life; if, in his intercourse with his friends, his words are sincere—although men say that he has not learned, I will certainly say that he has. (*Lunyu* 1.7).” (Legge, 1861)^25^.

Moreover, inasmuch as the process through which the *ren*-humane mind is realized or expressed in concrete *li*-proper social actions is a dynamic one, there are various ways in which it may be displayed or illustrated. Therefore, the ways of teaching which appear in the Confucian classics often tend to include a number of possible social actions as the empirical examples to be looked at, and the emphasis is on flexibility and open-mindedness. That is, the student should learn how to judge which mode of behavior will be best in any given social interaction (or any given situation). As the *Xueji* (*Lessons for Learning*) tells us: “In a superior man's (a sage's) teaching, he leads and does not drag; he strengthens and does not discourage; he opens the way but does not conduct to the end (without the learner's own efforts). Leading and not dragging produces harmony. Strengthening and not discouraging makes attainment easy. Opening the way and not conducting to the end makes (the learner) thoughtful” (Legge, 1885)^26^. This teaching and learning process is based upon the *ren*-benevolence of both teachers and leaners which, as a common bond, unites them. It gives the learners enough social space and inner reflection to cultivate their ideal Selves, where here we may think again of this Self in the light of Hwang's social Person and also his biological Individual.

### Hwang's Mandala model of the self and the confucian praxis of self-cultivation

The self, of course, needs to know how to be pragmatic, to take action. As the *Doctrine of the Mean* (*Zhong Yong*) says: “*Zhi*-knowledge/wisdom,*ren*-humanity/ benevolence, and *yong*-bravery, these three are the virtues universally binding. And the means by which they carry these duties into practice is singleness.” That is, these three virtues are embodied in a single social action.

According to the Confucian teaching, we need to know how to choose the right direction, the right course of action, the one that will serve best to make our lives meaningful. Yet if one sincerely reflects on one's own *ren*-mind, then one will be able, step by step, to perform those social actions that are most proper to her/himself. That is, we cannot know how to perform these actions only through our pre-existing embedment in society but must look into ourselves, into our own *ren*-minds, to know this. Therefore, our education must allow us to cultivate ourselves, must give us, or rather allow us to discover, a space within ourselves where or through which we can enrich our own lives, make them meaningful.

As for the art of teaching, Mengzi distinguishes these five levels:

There are five ways in which the superior man effects his teaching. There are some [students] on whom his influence descends like seasonable rain. There are some whose virtue he perfects, and some of whose talents he assists the development. There are some whose inquiries he answers. *There are some who privately cultivate and correct themselves*. These five ways are the methods in which the superior man effects his teaching (Legge, 1895)^27^.

The best teaching is thus like a seasonable rain which nourishes students, and more generally which nourishes all beings in the world so that they may grow naturally. Therefore, we may say that Confucian teaching is not only very compassionate but—like the rain that nourishes a fertile field—also very open and free. It cultivates one by giving one enough personal—and individual—space, enough *ren*-humanity or *ren*-benevolence, to self-reflect and transform one's inner knowledge and wisdom into a worthy and fulfilling social praxis.

### Hwang's model, meditation, confucianism

The Mandala Model of the Self gives us the interaction or “overlapping” of the Individual—who/which may include not only what he/she has learned about the outside world but also his/her innermost depths of thinking and reflection, perhaps even self-reflection or meditation—and the Person, who/which embodies the individual's participation in a larger group, a family or community, or society. As an active member of this larger group, one may choose a suitable job or career or other form of social action, based both on what he/she has already learned and on his/her ongoing experience of life in the world (in the society) (Eckensberger, 2012; Hwang, 2014, p. 42).

However, in order to make proper judgments and perform well in various social interactions, one may also need to learn how to transform his/her knowledge in the process of becoming-a-person. In the Confucian classics, which greatly influenced Hwang, we find a series of teaching and learning procedures whose purpose is to guide the self *to regulate its inner conflicts and its outer social relations* while maintaining a harmony between these two domains. As for the pursuit of knowledge, the Confucian teaching generally emphasized the hierarchal order of learning—moving from easier to more difficult subjects, from concrete life experiences to more abstract principles. In the *Xueji* (*Lessons for Learning*) of the *Liji* (*Classic of Rites*):

According to the system of ancient teaching, for the families of (a hamlet) there was the village school; for a neighborhood there was the *xiang* (a kind of school); for the larger districts there was the *xu* (bigger than a *xiang*); and in the capitals there was the college. Every year some entered the college, and every second year there was a comparative examination.…In the ninth year, when [the students] knew the different types of subjects, had gained a general intelligence (wisdom), and were firmly established and would not fall back, they were said to have made great attainments (Legge, 1885)^28^.

The above passage describes the hierarchical learning order, where the last step is the most difficult: the transformation of knowledge into wisdom. In fact, at this point the Confucian teaching particularly emphasizes “reflection.” There is a series of procedures for attaining wisdom step-by-step through self-reflection. In the *Great Learning* (*Daxue*) of the *Liji* (*Classic of Rites*), there is a paragraph describing this process in detail:

The point where to rest being known, the object of pursuit is then determined; and, that being determined, a calm unperturbedness may be attained to. To that calmness there will succeed a tranquil repose. In that repose there may be careful deliberation, and that deliberation will be followed by the attainment of the desired end. Things have their root and their branches. Affairs have their end and their beginning. To know what is first and what is last will lead near to what is taught in the *Great Learning* (*Da Hsueh*) (Legge, 1885)^29^.

Here we see the movement back to one's inner self or mind, to a state of tranquil repose—comparable perhaps to the Hindu or Buddhist meditative state—which makes possible “careful deliberation” and thus “the attainment of the desired end.”

After this transforming of his knowledge into wisdom, then, the student will be able to judge the rightness and wrongness of social actions. The *Doctrine of the Mean* (*Doctrine of Moderation*) (*Zhong Yong*) in the *Liji* (*Classic of Rites*) says: “He who attains to sincerity is he who chooses what is good, and firmly holds it fast. To this attainment there are requisite the extensive study of what is good, accurate inquiry about it, careful reflection on it, the clear discrimination of it, and the earnest practice of it” (Legge, 1885)^30^. That is, once one can carefully reflect and then clearly distinguish right and wrong, the next step is to sincerely practice what is right. If one can often do this, then he or she will have a happy life.

In terms of the Confucian *Analects* (*Lunyu*), this means that one (a student) with a *ren*-humane mind will naturally (spontaneously) know how to perform *li*-proper actions in the society. Confucian knowledge, then, is based on a moral orientation (ethical distinctions), and not—like cognitive knowledge—on the distinctions made by our senses, our sense perception. In fact Neo-Confucian scholars in the Song dynasty (960–1279 AD), such as Chang Zai or Cheng Hao, distinguished cognitive knowledge (*wen jian zhi zhi*, 
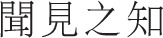
) from moral knowledge (*de xin zhi zhi*, 
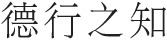
), and they also said that moral knowledge provides us with a foundation that is more general or universal than that provided by cognitive knowledge. In other words, we should first determine whether something we have learned or created *via* our senses has a moral meaning or purpose before we go ahead to base our actions on it^31^. Finally, then, our wisdom is grounded in our moral knowledge, and our primary goal is to transform our knowledge into wisdom, which is what guides our actions.

In fact, in ancient Confucian culture there are two main stages in the pursuit of self-cultivation: the “inner sage (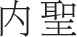)” and “outer kingdom (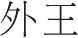).” The “inner sage” refers to the process of inner moral development, while the “outer kingdom” refers to the process of outward social development: here one's social development is regarded as the further fulfillment of his or her moral development. In the *Great Learning* (*Daxue*) of the *Liji* (*Classic of Rites*), we have these two stages: “Things being investigated, knowledge became complete. Their knowledge being complete, their thoughts were sincere. Their thoughts being sincere, their hearts were then rectified. Their hearts being rectified, their persons were cultivated” (Legge, 1885)^32^.

The above is seen as inner self-cultivation, which guides one to develop his/her own inner moral discrimination or moral knowledge to know what actions are right or wrong. However, only knowing what is right or wrong is not enough. One needs to further realize this moral knowledge by following the steps of “outer kingdom” to make the world better. These steps are found in the *Great Learning* (*Daxue*) of the *Liji* (*Classic of Rites*): “Their persons being cultivated, their families were regulated. Their families being regulated, their States were rightly governed. Their States being rightly governed, the whole kingdom was made tranquil and happy” (Legge, 1885)^33^.

This process of reaching the “outer kingdom” of self-cultivation could be regarded as moral-based social development and as “outer representation” in the Mandala model. Here, as the “inner sage” stage transforms into the “outer kingdom” stage, it needs wisdom to judge which is the most proper form of behavior or knowledge. Yet this transformation from “inner sage” to “outer kingdom” may also be understood as the transformation of inner self-reflection (of the reflective mind) into outer cognition (the outer cognitive mind).

### Mou's meditation/cognition opposition, his awareness of unexpected developments, and self-society-cosmos-*Tian*

Self-reflection or meditation is an inner operation of the mind which is peaceful, silent, beyond the subject-object distinction; in contrast, in cognitive learning the mind is oriented toward the outside world and needs the cognitive knowledge provided by the five senses. However, it is not so clear that Hwang's Mandala model of the self, which can clearly account for inward meditative thinking and outward social awareness or social thinking, can also account for or encompass cognitive thinking. The well-known Neo-Confucian scholar Zhongshan Mou, who also influenced Hwang, claims that self-reflection (or meditation) and cognitive learning are not only different mental operations, but that they are mutually exclusive–we cannot engage in both at the same time. Yet he thought that today one also needs scientific knowledge in order to fulfill his/her moral ideals. This means a self-transformation process that moves from inner self-reflection to the outer cognitive mind (Mou, 1980, pp. 44–62).

However, while Hwang was influenced by Mou in some ways, it is not clear that Hwang's Mandala model can enable, account for, serve as a foundation for the acquiring of empirical scientific knowledge. On the other hand, it is also true that Mou, who was influenced by Hegel as well as Kant, believed that no objectivity is possible apart from subjectivity, and he may seem to have ultimately emphasized subjectivity insofar as he believed in an ultimate reality (*benti*, 
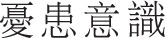
), and—like the classical Confucians—believed that human morality is ultimately validated by the “Way of Heaven”(*Tiandao*), the ultimate moral principle. Moreover, Mou claims that a person's uncomfortable reaction to crime and degeneracy indicates the existence of a moral consciousness, which he regards as the inner essence of human beings (Mou, 1990, pp. 37–56).

Coming back to Hwang and his Mandala model, while Inner Balance could clearly be correlated with, or be the goal of, meditation, it is not so clear that Outer Manifestation can be correlated with cognitive thinking/learning/knowledge. The correlation in the model of Knowledge/Wisdom with the Individual may also seem to suggest a priority to private, inward meditation, whose opposite or “other side” may not be cognitive thinking or learning but rather that same social harmony (via the Person) which the original Confucius would correlate with *Tian* (Heaven) and the *Tian Dao*. Thus, while it is clear that—as regards the Mandala model—individuals, families and communities today might want to “keep the whole model,” it is not so clear that cognitive and empirical subjects such as mathematics and the sciences could be included within it^34^.

Moreover, as for Ethics, it could be argued that the concept of “morality” in Chinese philosophy originates from what Zhongshan Mou calls “the awareness of unexpected developments, or existential anxiety” (*you huan yi shi*, 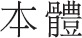)^35^. On the individual level the self is always already aware of its *temporal limitations*, for human life is short, temporally very limited within a vast and changing cosmos^36^, but now, as we are speaking of morality within the context of Neo-Confucian thought, we are already understanding this sense of life's contingency, uncertainty, finitude as it is experienced on a social level (Mou, 1990, pp. 16–18). The self as an Individual has to deal with individual needs and, as a Person, to deal with social needs, with the obligations and proper forms of behavior that arise with social relationships^37^. Here then the “Confucian awareness of unexpected developments” (*you huan yi shi*) can be seen as a form of anxiety experienced on both the individual and social level, but the latter is clearly seen by (Neo-) Confucianism as taking priority.

For one thing, while each self as an Individual and thus (in Hwang's model) as a biological entity may be concerned about physical (biochemical) survival, each self as a Person and thus as a member of society may be concerned with something that seems far more momentous—the survival or continuation of the whole society (which would also ensure his/her continuation as a Person). After all, it would be difficult for someone to create a meaningful life for him/herself if there seemed to be a lack of harmony or meaning or happiness in the whole society. It is also possible to see this the other way around, and say that each individual must be well-ordered if the whole society can itself be in a state of harmony. Thus, in a famous passage from the beginning of a Confucian Classic, *Daxue* (*Great Learning*), we read:

“Their persons being cultivated, their families were regulated. Their families being regulated, their states were rightly governed. Their states being rightly governed, the whole kingdom was made tranquil and happy. From the Son of Heaven down to the mass of the people, all must consider the cultivation of the person the root of everything besides. It cannot be, when the root is neglected, that what should spring from it will be well-ordered” (Legge, 1885).

Of course, if the “Individual” (in the model) inevitably suffers from anxiety regarding his/her own existence, then the Person may also suffer from a different kind of anxiety regarding the whole family or whole Society. If the actual life of the Individual is at stake, then so is the actual “life” of the Society—its well-being, harmony, ability to avoid war, and so continue to enjoy a Peaceful state^38^. A poor beggar may die of hunger in the streets of a city whose inhabitants do not care about him or her, just as a whole city may tend to disappear if all its citizens are being killed or starving to death in a terrible time of war and/or famine.

Both Hwang and Mou, who as Confucians have a solid background in classical Confucianism, of course stress the ultimate need for a sort of spiritual transcendence which goes beyond both the Individual and the Person, the self and society—and the need for this sort of transcendence might seem to be felt especially in times of social chaos, as for example, once again, in times of terrible war or famine. This giving of priority to a fully transcendent level, beyond both individuals (persons) and the whole society, tends to support the idea of the greater importance of Whole. Here we may think of religions like Buddhism, Hinduism, Daoism, and perhaps also Near-Eastern religions like Judaism, Christianity, and Islam. In fact, while the ancient Chinese conception of *Tian* (Heaven) implied at first the supernatural or religious power of Fate (the *Tian Ming*, Decree of Heaven) and then the power of political forces (“imperial decrees”), it had become for Confucius, in the sixth-century AD, an ideal that was at once personal, social and fully transcendent, that is, in some sense “divine.” The individual, his society and his ancestors were all seen as a manifestation of *Tian*.

*Tian* for Confucius in the later Zhou dynasty was thus paradoxically both an immanent and a transcendent concept, for now everyone had the right to receive the “Decree of Heaven.” That is, if one could truly understand and appreciate what *Tian* had conferred upon him—his essentially good human Nature, his Self—and could then go on to fulfill—through his social actions and social relationships—his own potential, his own true Nature in the society—we could say he was following the will or spirit of Heaven. As Confucius said, *Tian* gives birth to all things on earth equally and yet lets each one of them “arise differently” (Wu, 2015), Thinking in terms of Hwang's Mandala model, then, we could say that once the Self can regulate *its inner conflicts and its outer social relations*, transforming its moral knowledge into wisdom, then it/she/he will be able to completely develop and make use of his/her talents in the society as well, in a certain sense, as in the cosmos. We could say that this person has then fully realized within him/herself—and also has seen or understood as being the “decree of Heaven”—that which Hwang tries to encapsulate with, within and through his Mandala model.

## Conclusion

Kwang-Kuo Hwang, in his Mandala model of the self, is influenced by the ancient Chinese Confucian conception of human life as an open field, so that our cultivating of this field is really our own self-cultivation—for after all, our self was formed out of this wider field and as such it (our self) can still be further cultivated. We will also recall that, for Hwang, the Person (at the top of the circle) belongs to the sociological domain, playing various social roles within society, while the Individual (at the bottom) belongs to a purely biological and physical level as an individual being within nature. Correlated with Person and Action/Praxis on the top and on the right, we also have “outer representation” in the top right corner of the square; correlated with Individual and Knowledge/Wisdom on the bottom and on the left, we also have “inner balance” in the bottom left corner. As for the praxis of “cultivating the self or Self,” this is of course the function of schools, of education.

Here it is clear that Hwang's Mandala model may capture or express the basic framework, contours, vectors according to which one may fulfill or realize his/her need for self-understanding, both as a private Individual and a social Person, through both inward meditative thinking and outward social engagement, social action. After all, the pictorial form of the model suggests a series of overlapping *fields to be cultivated*. Here we might think first of *self-cultivation*, but may also think of “education” which comes from the Latin *educare*—to draw out, bring up, rear, raise (a child). Self-cultivation (self-education) is one thing, but schools are places that “cultivate” (educate) young people, teaching them various subjects but most fundamentally, perhaps, helping them to develop (cultivate) themselves, to grow, both as private Individuals and as social Persons, where the “sociality” would already seem to imply “as moral Persons.”

Zhongshan Mou, in addition to his conception of the “self-negation of consciousness” (*lian zhi zhi zi wou kan xian*), emphasizes “the awareness of unexpected developments” (*you huan yi shi*), that is, the Individual's awareness of the spatial and temporal limitations of his/her own life, his/her own finitude, and the utter unpredictability of life, of the future. Yet this awareness may well force the Individual to choose which courses of action to take in life, to determine which actions would be moral, wise, humane, socially correct, and socially beneficial—in other words, force him to become (also) a Person. To be an ideal modern person from the (Neo-)Confucian perspective, one may need to follow two paths—to gain an objective understanding (through perception and objective conscious awareness) of one's role, one position, or location in the world (as Individual and person), and also (through an emptying of the mind) to follow the Tian Dao, Way of Heaven.

Thus, though (Neo-)Confucianism may seem at first to give a priority to moral knowledge (or wisdom) over purely sensory or cognitive knowledge, and to the society over the individual, its core concern is really the interrelationship of individual and society, of knowledge (wisdom), and action (praxis)—as should already be clear from Hwang's model. Moreover, communities, societies, and nations themselves tend also to be transient in the long run, and to experience “existential anxiety” about their own (in)stability, their (im)permanence^39^. We can see how the individual's awareness of life's finitude and of the inevitability of death, his/her feeling that our own short lives are finally meaningless within the vast universe of space and immense ocean of time, could suggest a perspective closer to that of Hinduism, Buddhism, and perhaps Daoism than to that of Confucianism.

Yet the latter has had, since the late Shang and early Zhou dynasties (circa 1000–1200 BC), the conception of *Tian* (Heaven) and the *Tian Ming* (Decree of Heaven). This at first seemed mainly to represent the emperor's absolute power but then gradually came to represent, and very clearly by the time of Confucius in the 500's BC, something much closer to a transcendent moral ideal that was present in each individual and also embodied in the whole community, society, empire. Thus, perhaps this transcendent moral, individual and also communal ideal of *Tian* might just embody the fusion of individual/person, wisdom/action, inner/outer, immanent/transcendent. For Confucius himself, who accepted young men from all social classes as his students, the fusion of self/other, the *ren*-humane putting of oneself in the other one's shoes (so that in a sense the duality disappeared) was of course the essential paradox and the very embodiment of wisdom. For him the concept or ideal of *Tian* also captured this idea, this paradox.

## Author contributions

The author confirms being the sole contributor of this work and approved it for publication.

## Funding

This research is supported by Taiwan's Ministry of Science and Technology (grant number: MOST 105-2410-H-017-023).

### Conflict of interest statement

The author declares that the research was conducted in the absence of any commercial or financial relationships that could be construed as a potential conflict of interest.

## Footnotes

^2^For Plato, Aristotle, Descartes, Hume, and Kant, see Frederick Copleston (1962).

^3^The German term “*Ich*” is normally translated into English as “I,” “the self,” “ego.” In Freudian research it is translated as “ego.”

^4^According to the etymology, the term of *Jia* () means that there are farm animals living under the house, and human beings living in or “above” the house. The term *Ting* () refers to human beings' activities in front of or under the building.

^5^According to the etymology, the term for benevolence, *ren* (), means two persons, i.e., the interaction between person and person.

^6^In *tan-chang*, the left side of the character *tan* () means or pictures the soil or ground, and its right side pictures/means to clean a space, a field where a ceremony like that of giving sacrifices to the ancestors or gods can be held; the character *chang* () means a place or area.

^7^In fact, Huang was much influenced by indigenous studies and by the anthropologist Grace Harris (who had originally proposed the individual/person/self-concept). Like Harris, he thought that while all non-western (including East Asian) cultures share with indigenous cultures an emphasis on the inter- connectedness of people and the community, so that individuals also tend to already be or will easily become members of the group or social “persons,” western cultures tend to focus too much on the individual which they see “as a member of the human species, not so different from other creatures in the universe.” For Huang, within the conceptual framework of the Figure 1, the Self is the locus of experience and as such is able to unite the roles of Individual and Person, interconnect various actions in different social contexts, and engage in self-reflection when blocked from attaining its goals. The Individual tends to have a more inward focus, while the Person takes a certain standpoint in the social order and plans a series of actions to attain a certain goal. Hwang was also much influenced by the French psychologist Piaget, whose theory of childhood development uses the core concepts of personal and social knowledge, and by the French sociologist Bourdieu's constructivist structuralism; the latter's term “*habitus*” denoted a kind of “structuralized behavioral tendency” (Harris, 1989; Hwang, 2011).

^8^Regarding cultural inclusion theories and the concepts of cultural morphostasis and cultural morphogenesis, please also see Archer (1995) and Hwang (2015, p. 38).

^9^Regarding the *Ren*-*Li* issue in the *Analects*, please see Wu (2013).

^10^According to the author's analysis of the *Analects* and *Mengzi*, the four terms *Ren, Yi, Li*, and *Zhi* only appear together in the *Mengzi* 6 times, while they cannot not be found together in the *Analects*. In the latter, only *ren* and *li* often appear in the same passage. Please see Wu (2013).

^11^The relevant document retrieved from http://ctext.org/liji/li-yun.

^12^Mengzi used a well-known example to show this. For the relevant explanation, please see Wu (2013).

^13^The relevant document was retrieved from: http://ctext.org/liji/zhong-yong.

^14^Retrieved from: http://ctext.org/liji/jing-jie

^15^Retrieved from: http://ctext.org/analects/ba-yi

^16^Retrieved from: http://ctext.org/analects/wei-zheng

^17^Retrieved from: http://ctext.org/liji/yue-ji

^18^Retrieved from: http://ctext.org/analects/tai-bo

^19^Retrieved from: http://ctext.org/analects/yong-ye

^20^Retrieved from: http://ctext.org/analects/li-ren

^21^For a more detailed account of the relevant discussion see (Wu, 2016).

^22^Retrieved from: http://ctext.org/liji/xue-ji

^23^Retrieved from: http://ctext.org/liji/xue-ji

^24^Retrieved from: http://ctext.org/liji/xue-ji

^25^Retrieved from: http://ctext.org/analects/xue-er

^26^Retrieved from: http://ctext.org/liji/xue-ji

^27^Retrieved from: http://ctext.org/mengzi/jin-xin-i

^28^Retrieved from: http://ctext.org/liji/xue-ji

^29^Retrieved from: http://ctext.org/liji/da-xue

^30^Retrieved from: http://ctext.org/liji/zhong-yong

^31^Obvious examples are cases involving the use of atomic bombs in war (or even the testing of them on remote Pacific islands), or more recently issues regarding national security that have arisen from the creation of the Internet. These inventions were, of course, a product of the physical sciences.

^32^Retrieved from: http://ctext.org/liji/da-xue

^33^Retrieved from: http://ctext.org/liji/da-xue

^34^However, if we consider Huang's discussion of cultural inclusion theories and the concepts of cultural morphostasis and cultural morphogenesis, then, we can say that the Mandala model is a universal model which can be applied to various cultural systems. If we apply Piaget's concept of adaptation, which is related to the concepts of assimilation and accommodation, to a developing cultural system, we may say that once a cultural system cannot adapt to a new experience, such as western cognitive thinking, by assimilation, then it may develop its schema by means of accommodation, that is, by absorbing new cultural experiences. For example, some Confucian scholars in modern China and Japan suffered from western aggression beginning in the nineteenth century; therefore (as we know from a series of documents) they set out to acquire cognitive, even scientific knowledge in order to save their countries. Here their desire to absorb western knowledge could be regarded as a moral decision to pursue self-cultivation, and thereby transform (their) inner transcendence into outer transcendence, fulfilling the ideal Self. For the relevant research, please see Hwang (2015, p. 38); and Wu (2009, pp. 309–328).

^35^This anxiety may be similar to that of Heidegger (*angst*) and existentialism, except that now we are already looking at this awareness and this anxiety as they are experienced by the whole society.

^36^Laozi said: “What makes me liable to great calamity is my having a body (which I call myself); if I had not the body, what great calamity could come to me? (Laozi Ch. 13)” (Legge, 1891).

^37^For further discussion of the time-engaged self as individual and as social self, see also Cheng (2004). Regarding the relevant arguments, please see also Wang et al. (1989, pp. 1–78.) In Chapter 1, entitled “the ideal of human life,” Wang claims that as one is aware that human life has an objective limit, he/she needs to undertake a series of self-cultivation processes to realize his/her ideal Self. In Chapter 2, “The limitations of our existing life,” Zheng discusses how (Neo-)Confucian scholars overcame various difficulties to extend their limited physical lives into permanent spiritual ones.

^38^Here one might think of the mass anxiety felt by Clinton supporters, and the widespread rioting in the streets—threatening to create disorder throughout the whole society and put its own peaceful and orderly future at risk—in the wake of Trump's winning of the recent presidential election in the USA.

^39^We may also think about this anxiety, uncertainty, *disorder* in the realm of political power. “When looking at society we see the importance of the family;…even states in China can be viewed as the family writ large. There is an overall orientation toward conciliation and harmony and a deep-seated anxiety of disorder (*luan*). Even the Confucian golden rule [do not do unto others as you would not have them do unto you] is predicated on the notion that order should prevail, that disorder is dangerous, and hence that discipline is necessary” (Tu, 1992, pp. 17–18). Here this golden rule is implicitly being contrasted with the “positive” western one: “Do unto others as you would have them do unto you.”
